# Novel peptides suppress VEGFR-3 activity and antagonize VEGFR-3-mediated oncogenic effects

**DOI:** 10.18632/oncotarget.1709

**Published:** 2014-05-24

**Authors:** Yi-Wen Chang, Chih-Ming Su, Yen-Hao Su, Yuan-Soon Ho, Hui-Huang Lai, Hsin-An Chen, Min-Liang Kuo, Wen-Chun Hung, Ya-Wen Chen, Chih-Hsiung Wu, Pai-Sheng Chen, Jen-Liang Su

**Affiliations:** ^1^ Graduate Institute of Biochemistry and Molecular Biology, National Yang-Ming University, Taipei 112, Taiwan; ^2^ Genomics Research Center, Academia Sinica, Taipei 115, Taiwan; ^3^ National Institute of Cancer Research, National Health Research Institutes, Miaoli 35053, Taiwan; ^4^ Graduate Institute of Clinical Medicine, College of Medicine, Taipei Medical University, Taipei 110, Taiwan; ^5^ Division of General Surgery, Department of Surgery, Shuang Ho Hospital, Taipei Medical University; ^6^ School of Medical Laboratory Science and Biotechnology, College of Medical Science and Technology, Taipei Medical University; ^7^ Institute of Basic Medical Sciences, National Cheng Kung University, Tainan 701, Taiwan; ^8^ College of life Science, National Taiwan University, Taipei 106, Taiwan; ^9^ Department of Medical Laboratory Science and Biotechnology, National Cheng Kung University, Tainan 701, Taiwan; ^10^ Graduate Institute of Cancer Biology, College of Medicine, China Medical University, Taichung 404, Taiwan; ^11^ Center for Molecular Medicine, China Medical University Hospital, Taichung 404, Taiwan; ^12^ Department of Biotechnology, Asia University, Taichung 404, Taiwan

**Keywords:** VEGFR-3, VEGFR-3-targeting peptides, metastasis, drug resistance

## Abstract

Vascular endothelial growth factor receptor 3 (VEGFR-3) supports tumor lymphangiogenesis. It was originally identified as a lymphangiogenic factor expressed in lymphatic endothelial cells. VEGFR-3 was detected in advanced human malignancies and correlated with poor prognosis. Our previous studies show that activation of the VEGF-C/VEGFR-3 axis promotes cancer metastasis and is associated with clinical progression in patients with lung cancer, indicating that VEGFR-3 is a potential target for cancer therapy. In this study, we developed eight peptides targeting VEGFR-3. Two peptides strongly inhibited the kinase activity of VEGFR-3 and suppressed VEGF-C-mediated invasion of cancer cells. Moreover, these peptides abolished VEGF-C-induced drug resistance and tumor initiating cell formation. This study demonstrates the therapeutic potential of VEGFR-3-targeting peptides.

## INTRODUCTION

Over the past 20 years, cancer cell surface receptors have been successfully used as molecular targets for cancer therapy and diagnosis because the receptors are typically overexpressed in tumors relative to normal tissues [1]. Although efforts to develop small molecules, such as sunitinib and sorafenib, are ongoing, most of these chemical agents have a narrow therapeutic window because of serious toxicity to normal tissues. Therapeutic peptides with low toxicity and high specificity are therefore one of the most promising options for cancer treatment because of they are of low molecular weight and are easily synthesized.

Receptor tyrosine kinases (RTKs) were the first kinases to be linked to tumorigenesis and stand as ideal targets for the development of targeted cancer therapies. Agents targeting RTKs in cancer include small molecules, therapeutic antibodies, and blocking peptides. Monoclonal antibodies to VEGFR-1 block tumor migration, invasion and colony formation in vitro in VEGFR-1-expressing human colon cancer cells [2]. More recently, Wu et al. demonstrated that an anti-VEGFR-1 monoclonal antibody abolished its signal transduction and the subsequent growth of human breast tumors [3]. Furthermore, a phase II clinical trial suggested that treatment with bevacizumab attenuated the activation of VEGFR-2 in human breast tumors [4]. These studies indicate that VEGFRs are ideal targets for the development of therapeutic agents because they are overexpressed and functional in tumor cells.

The VEGF-C/VEGFR-3 axis has been documented as a gatekeeper signaling pathway for triggering lymphangiogenesis, which is an important step in tumor progression. The activation of VEGFR-3 induces the formation of lymphatic vessels within and around tumors and enhances metastatic spread via the lymphatics [2, 5, 6]. Similar to angiogenesis, a tumor can induce its own network of lymphatics that connect to the surrounding lymphatic vessels. Indeed, clinical and pathological observations suggest that for many carcinomas, the transport of tumor cells by lymphatics is the most common pathway of initial dissemination, such that cancers spread by afferent lymphatics that follow natural routes of drainage [7]. In addition to its role in lymphangiogenesis, the expression of VEGFR-3 has been correlated with poor survival in colon cancer [8]. VEGFR-3 expression has also been correlated with advanced stages of cervical carcinogenesis [9]. Moreover, the activation of the VEGF-C/VEGFR-3 axis was shown to protect leukemic cells from the apoptotic effects of chemotherapeutic agents such as cytarabine, doxorubicin and etoposide [10]. Our previous studies provided evidence that the VEGF-C/VEGFR-3 axis enhances cancer cell mobility and invasiveness, contributes to the promotion of cancer metastasis, and is closely correlated with clinical metastasis and survival in patients with lung cancer [11]. We also found that blocking the VEGF-C/VEGFR-3 axis dramatically decreased cancer metastasis in an animal model [11]. These results strongly suggest that VEGFR-3 is a crucial RTK and that its therapeutic potential strongly merits further investigation.

## RESULTS

### Identification of peptide sequences that bind to human VEGFR-3

Our previous studies [11, 12] suggested that the identification of VEGFR-3-targeting peptides for cancer treatment is important and highly feasible. To identify peptides that bind VEGFR-3, we established a phage display technique to screen a 7-mer random cyclic peptide phage library. A random 7-mer peptide phage library composed of 2 × 10^9^ independent phage clones was obtained from a biopanning screen against plate-bound soluble VEGFR-3 (sR3). The VEGFR-3-bound phages were recovered by elution with acidic buffer. For each biopanning, the number of phages (pfu) in the inputs and outputs were compared to determine the degree of selection. Eight phage clones were identified for further studies based on their VEGFR-3 binding affinity (Table 1). Supplemental Figure S1 shows the structure and residues of each peptide.

**Table 1:** Sequence of the peptides selected by binding to sVEGFR-3

Peptide Id	Peptide Sequence
P1	CPRSPSLTC
P2	CPRSPSLQC
P3	CNDSHMLHC
P4	CNDSHMKLC
P5	CNYSHLFYC
P6	CYYGQSKYC
P7	CYYWQSKYC
P8	CLTSSLRSC

### VEGFR-3-targeting peptides block the interaction between VEGF-C and VEGFR-3 and the inhibit kinase activity of VEGFR-3

**Figure 1:**
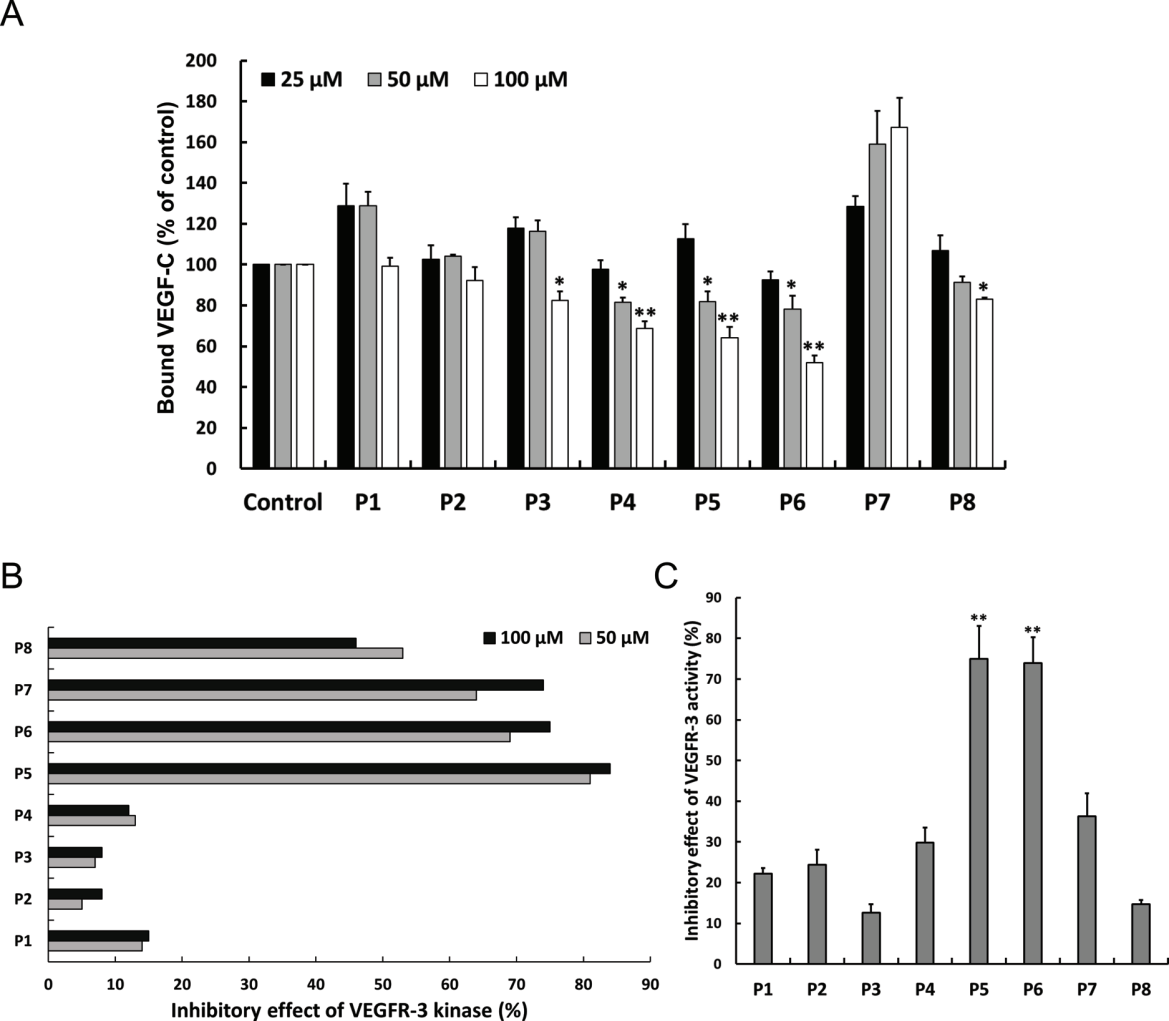
Effects of candidate peptides on antagonizing VEGFR-3 (**A**) Peptide competition assay were performed to analyze the competitive ability of indicated peptides to VEGF-C on VEGFR-3 binding. The indicated peptides were co-treated with VEGF-C to sVEGFR-3-bound plate. The level of sVEGFR-3-bound VEGF-C was measured by ELISA assay. *Columns*, mean of three independent experiments; bars, SD. *, *p* < 0.05; **, *p* < 0.01 statistically significant decrease compared with the corresponding control value. (**B**) The effect of peptides on VEGFR-3 tyrosine kinase activity by KIRA-ELISA assay. Tyrosine kinase activity of VEGFR-3 was analyzed by VEGFR-3 kinase assay as described in Materials and Methods. (**C**) Inhibitory effect of peptides on VEGFR-3 kinase activity. H928 cells were cultured in 24-well plate and then serum-starved overnight. Cells were pretreated with indicated peptides before rhVEGF-C treatment. The cell lysates were harvested and applied to VEGFR-3 coated ELISA plate. The level of phosphorylated VEGFR-3 was measured by KIRA-ELISA. *Columns*, mean of three independent experiments; bars, SD. **, *p* < 0.01 statistically significant increase compared with the corresponding control value.

To examine the ability and the affinity of selected peptides to bind sVEGFR-3, an *in vitro* competition assay was performed. These peptides were added to sVEGFR-3-coated wells to compete with VEGF-C binding, and bound VEGF-C was detected by ELISA. The P4, P5 and P6 peptides reduced the levels of bound VEGF-C in a significant and dose-dependent manner, whereas no obvious change was found among other peptides (Figure 1A). This result indicates that the P4, P5 and P6 peptides block the binding of VEGF-C to sVEGFR-3 and suggests that these VEGFR-3-binding peptides may affect the activation of VEGFR-3. To investigate the inhibitory effects of these candidate peptides on VEGFR-3 activity, we subjected these peptides to pan-lab (Ricerca Lab) to measure VEGFR-3 kinase activity. The P5, P6, P7 and P8 peptides exhibited the highest inhibitory effects on VEGFR-3 kinase activity (82% for P5, 72% for P6, 69% for P7 and 49% for P8) (Figure 1B). Moreover, we also analyzed the effects of these peptides on VEGFR-3 activity by kinase receptor activation enzyme-linked immunosorbent assay (KIRA-ELISA) [13]. The candidate peptides were pre-incubated with VEGF-C-treated H928 lung cancer cells, and whole-cell lysates were harvested to determine tyr-phosphorylated VEGFR-3 by KIRA-ELISA. The P5 and P6 peptides consistently showed the highest inhibitory effects on the phosphorylation of VEGFR-3 (Figure 1C). These results clearly show that the P5 and P6 peptides bind to VEGFR-3 and reduce the activity of VEGFR-3.

### The P5 and P6 peptides inhibit VEGFR-C-induced VEGFR-3 phosphorylation and the VEGF-C/VEGFR-3-mediated signaling pathway

**Figure 2:**
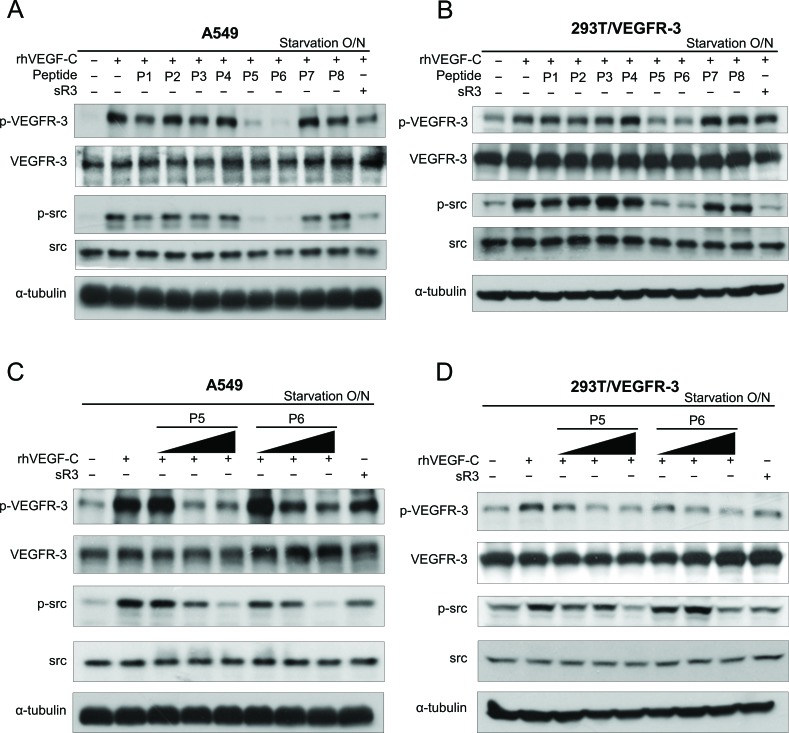
The effect of candidate peptides on VEGFR-3 phosphorylation and VEGFR-3-mediated signaling pathway (**A** and **B**) Effects of candidate peptides on VEGFR-3-mediated signaling pathway. A549 (**A**) and HEK293T/VEGFR-3 (**B**) cells were treated with rhVEGF-C in the absence or presence of candidate peptides or soluble VEGFR-3 (sR3; as the inhibitor for VEGF-C-induced VEGFR-3 activation). (**C** and **D**) Dose-dependent effects of P5 or P6 peptides on VEGFR-3-mediated signaling pathway. A549 (**C**) and HEK293T/VEGFR-3 (**D**) cells were stimulated with rhVEGF-C with increased concentrations of P5 or P6 peptides. Cell lysates were harvested and subjected to SDS-PAGE followed by western blot analysis with anti–p-tyr1063/1068-VEGFR-3 and anti–p-src antibodies.

It has been reported that tyrosine residues 1063 and 1068 (Tyr1063/1068) in VEGFR-3 enhance VEGFR-3 activation and function [14, 15]. We further confirmed the suppressive effects of the candidate peptides on VEGFR-3 phosphorylation. To test their effects on the activation of the VEGF-C/VEGFR-3 axis, A549 lung cancer cells with endogenous VEGFR-3 expression or 293T cells with ectopic VEGFR-3 expression (293T/VEGFR-3) were treated with peptides for 24 hours and then assayed to determine VEGF-C-induced VEGFR-3 Tyr1063/1068 phosphorylation. Both the P5 and P6 peptides exhibited dramatic suppressive effects on VEGFR-3 phosphorylation in A549 and human embryonic kidney 293T cells (Figure 2A and 2B). In our previous study, we identified that the VEGF-C/VEGFR-3 axis-mediated invasion of human cancer cells required the activation of the Src-p38-C/EBP-dependent pathway [11]. To investigate whether the peptides inhibited the VEGFR-3-mediated signaling pathway, we also determined the effects of these peptides on Src phosphorylation. Consistent with the patterns of phospho-VEGFR-3, decreased phospho-Src levels were found in the P5 and P6 peptide-treated A549 and 293T/VEGFR-3 cells (Figure 2A and 2B). Furthermore, a dose-dependent decrease in phospho-VEGFR-3 and phospho-Src were also observed in cells that were treated with increasing doses of the P5 and P6 peptides (Figure 2C and 2D). These results confirm that the P5 and P6 peptides are the most effective candidates among these peptides that can block VEGFR-3 activation and suppress its downstream signaling pathway.

### The P5 and P6 peptides suppress VEGF-C-induced migration, invasion and drug resistance in cancer cells

**Figure 3:**
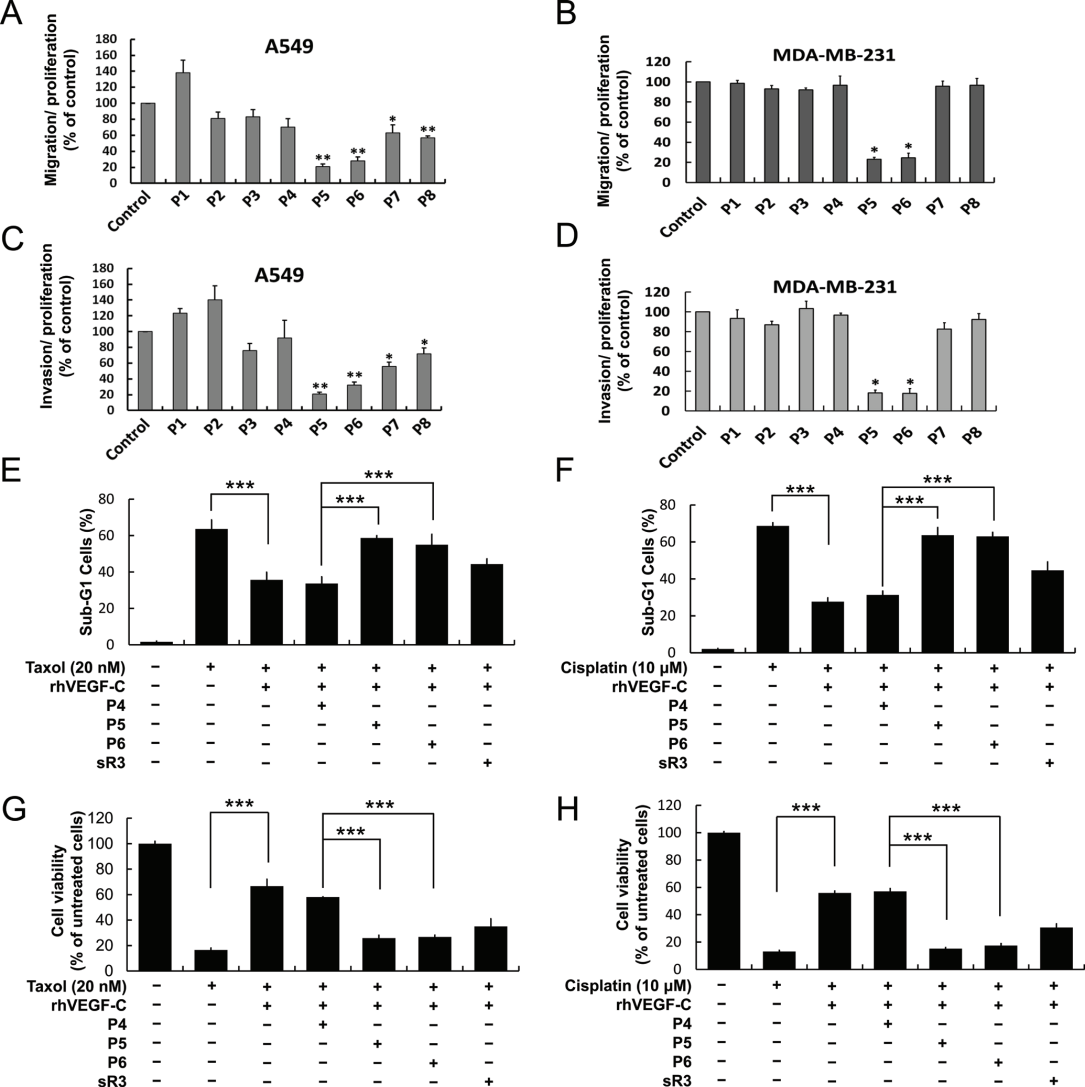
P5 and P6 peptides inhibit cancer cell migration, invasion and VEGF-C-induced drug resistance (**A** and **B**) Effects of candidate peptides on cancer cell migration and invasion. A549 and MDA-MB-231 cells were treated with indicates peptides and subjected to measure their migration (**A** and **B**) and invasion (**C** and **D**) abilities by modified Boyden chamber assay. (E-H) Effects of control (P4), P5 and P6 peptides on VEGF-C-enhanced drug resistance. Taxol and cisplatin were treated in A549 cells with the indicated peptides and rhVEGF-C. Cells were harvested and stained for DNA with Propidium iodide, flow cytometric assay was performed as described in “Materials and Methods” (**E, F**); the cell viability were measured by trypan blue exclusion assay (**G, H**) *Columns*, mean of three independent experiments; bars, SD. *, *p* < 0.05; **, *p* < 0.01; ***, *p* < 0.001 statistically significant decrease compared with the corresponding control value.

Previous studies indicated that VEGF-C promoted cancer cell survival, proliferation and metastasis [11, 12, 16]. Because the P5 and P6 peptides showed a stronger potential to inhibit VEGFR-3 activity, we next focused on determining their effects on cancer cell migration and invasion. A549 lung cancer cells and MDA-MB-231 breast cancer cells with endogenous VEGFR-3/VEGF-C expression were treated with the peptides and then evaluated for migration and invasion abilities by using the Boyden chamber assay. Obvious decreases in migration and invasion were observed in the P5 and P6 peptide-treated A549 and MDA-MB-231 cells (Figure 3A-3D). This evidence suggests that the P5 and P6 peptides not only suppressed VEGFR-3 activation but also subsequently diminished cancer cell migration and invasion. It has been reported that VEGF-C helps cancer cells to survive Taxol treatment via downregulation of mTORC1 function in prostate and pancreatic cancers [17]; additionally, VEGF-C was shown to enhance cisplatin-resistance through NF-κB activation in gastric cancer [18]. Moreover, VEGF-C-induced Akt activation has been suggested to promote prostate cancer cell survival [19]. Depletion of VEGF-C has further been shown to inhibit cell proliferation and cell invasion in non-small cell lung cancer (NSCLC) [20]. VEGFR-3 overexpression or activation also confers chemo-resistance to treatment in leukemia cancer by enhancing Bcl-2 expression [10]. We further assessed whether VEGFR-3-targeting peptides promote cancer cell chemosensitization. First, recombinant human VEGF-C (rhVEGF-C)-treated A549 cells were exposed to Taxol or cisplatin to investigate the functional role of VEGF-C/VEGFR-3 in drug resistance. We used DNA flow cytometric assays, trypan blue exclusion and MTT assays to detect the effects of VEGF-C on drug resistance in A549 cells. The cell viability of Taxol and cisplatin-induced cell death with different concentrations of VEGF-C was therefore tested (Supplemental Figure S2). Treatment with rh-VEGF-C significantly increased cell survival in the presence of Taxol or cisplatin, which indicated that the VEGF-C/VEGFR-3 axis plays a protective role when cancer cells are exposed to anti-cancer drugs (Supplemental Figure S2). To further confirm whether these peptides could block VEGF-C/VEGFR-3-mediated chemoresistance, these rhVEGF-C-treated cells were pre-incubated with the peptides and then flow cytometry and trypan blue exclusion assays were performed. Interestingly, the VEGFR-3-targeting peptides significantly abolished rhVEGF-C-induced drug resistance in A549 cells as measured by both the flow cytometric and trypan blue exclusion assays (Figure 3E-3H) and an MTT assay (Supplemental Figure S3).

### The P5 and P6 peptides decrease VEGF-C/VEGFR-3 signaling, cell mobility and cancer stemness

**Figure 4:**
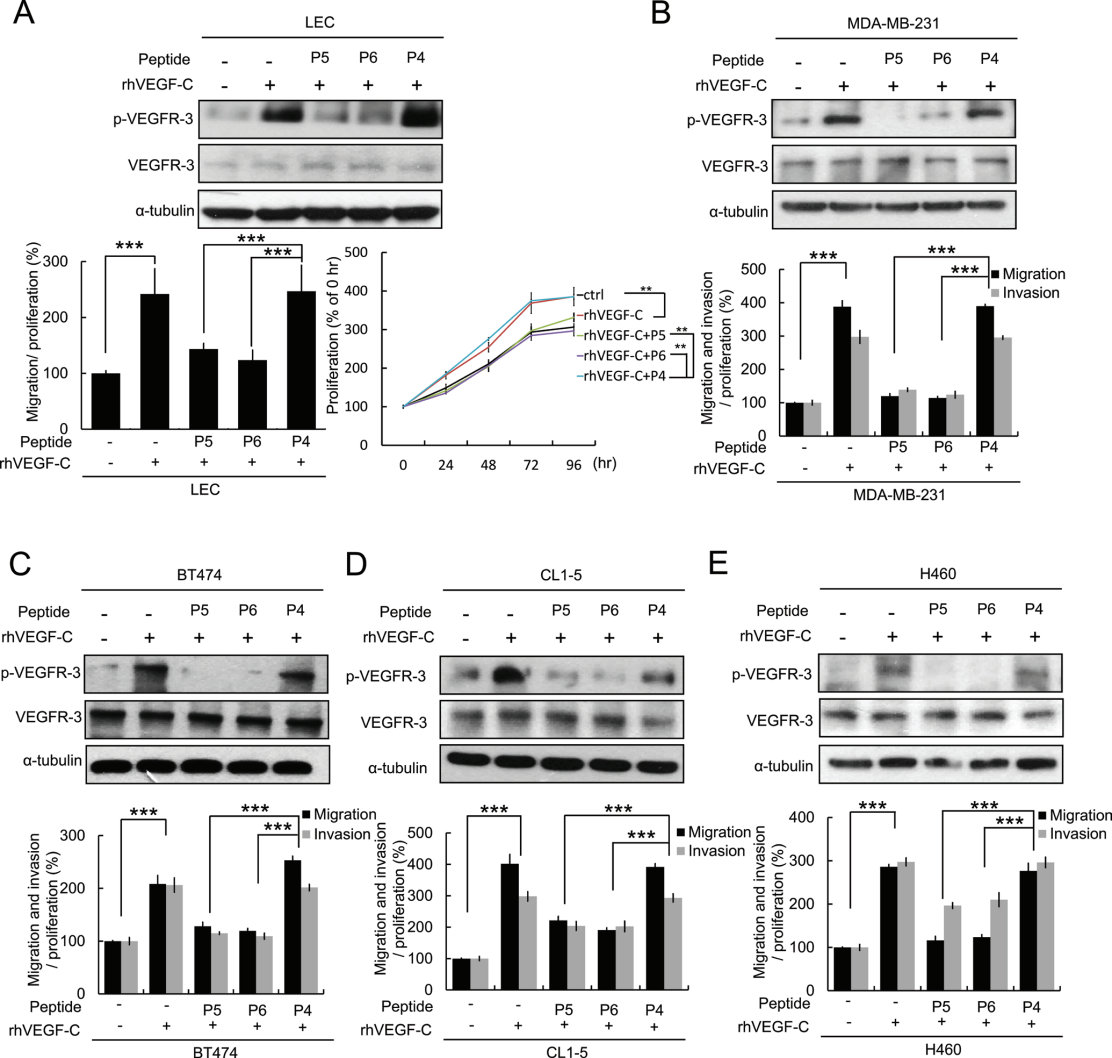
P5 and P6 peptides inhibit VEGF-C/VEGFR-3 signaling and cell mobility in LECs and multiple cancer cells (**A**) Effects of P4, P5 and P6 peptides on VEGF-C-induced VEGFR-3 phosphorylation, migration and proliferation in LECs were analyzed by western blot analysis, Boyden chamber assay and MTT assay. The statistically significant in the MTT assay mean compared with the indicated group at 96 hrs. (**B-E**) Effects of P4, P5 and P6 peptides on VEGF-C-induced VEGFR-3 phosphorylation was analyze by western blot analysis (upper) and the ability of migration and invasion (lower) in cancer cells. MDA-MB-231 (**B**), BT474 (**C**), CL1-5 (**D**) and H460 (**E**). *Columns*, mean of three independent experiments; bars, SD. **, *p* < 0.01; ***, *p* < 0.001 statistically significant decrease compared with the corresponding control value.

The VEGF-C receptor VEGFR-3 (FLT-4) is predominantly expressed by lymphatic endothelia in normal adult human tissues [21]. VEGF-C has been suggested to promote the proliferation of lymphatic endothelia cells (LECs) and to be a molecular link between tumor lymphangiogenesis and metastasis [6]. We were therefore interested in studying whether the P5 and P6 peptides inhibit VEGF-C/VEGFR-3 signaling and cell migration and proliferation in LECs. Our data indicated that VEGF-C-induced VEGFR-3 phosphorylation, cell migration and proliferation of LECs were abolished by the P5 and P6 peptides (Figure 4A). We further determined whether the P5 and P6 peptides inhibited VEGFR-3 phosphorylation, cell migration and invasion in various cancer cell lines (breast cancer cell lines: MDA-MB-231, BT474, HS578T, HBL100, MDA-MB-361, and MCF-7; lung cancer cell lines: CL1-5, H460 and H322) (Figure 4B-4E and Supplemental Figure S4). Our results indicated that the P5 and P6 peptides significantly inhibited VEGF-C-induced VEGFR-3 phosphorylation, cell migration and invasion in various cancer cell lines. Moreover, tumor-initiating cells (TICs) play important roles in regulating tumorigenesis, metastasis, and resistance to chemotherapy [22, 23]. It has been reported that down-regulation of VEGF-C decreases the TIC features of A549 cells [24]. Because VEGF-C was able to protect cancer cells from chemotherapy and was also necessary for maintaining TICs, we first investigated whether VEGF-C expression could increase the population of TICs. A549 and MCF-7 cells were incubated under conditions that favor cancer stem cell growth and then analyzed for their sphere-forming capacity representing their tumor-initiating phenotypes. Our results showed that rhVEGF-C treatment dramatically increased sphere number and size, which suggests an increase of TICs in both cell lines (Supplemental Figure S5). To further determine whether VEGFR-3-targeting peptides could abrogate VEGF-C-induced TIC formation, the P5 and P6 peptides were co-treated in the presence of rhVEGF-C. The treatment of the P5 peptide decreased the number and size of the spheres that were enhanced by VEGF-C in A549 and MCF-7 cells (Supplemental Figure S5A and S5B). Both peptides decreased the expression of *SOX2* (Supplemental Figure S6), which has been documented as a molecular feature of TICs [25, 26]. These data indicate another function of the VEGFR-3-targeting peptides in regulating TIC formation.

### The P5 and P6 peptides suppress VEGF-C-induced metastasis in an animal model

**Figure 5:**
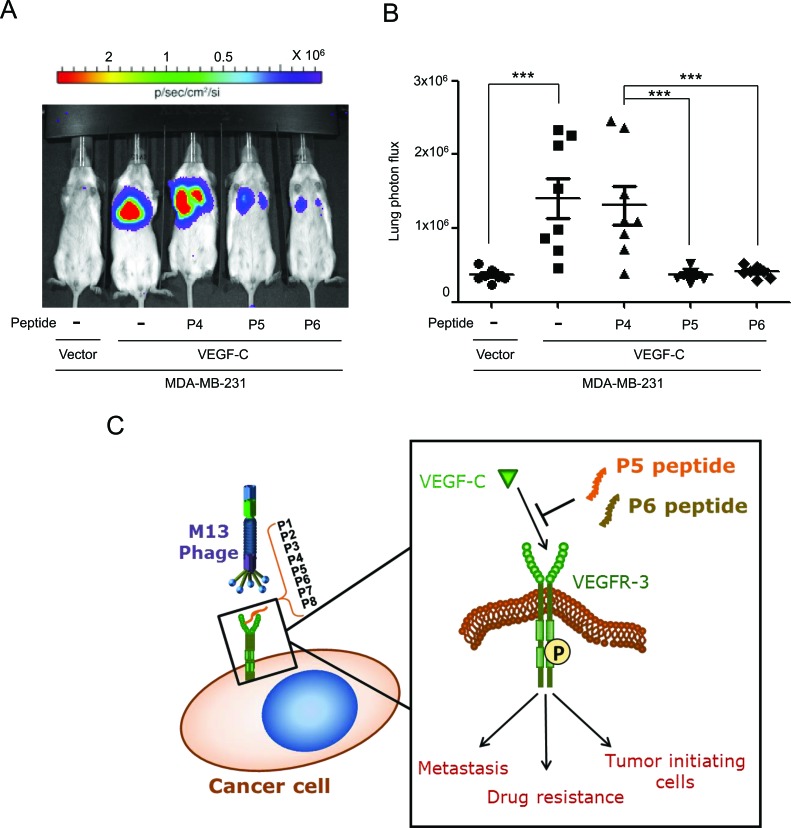
P5 and P6 peptides inhibit metastasis in an experimental metastasis animal model (**A** and **B**) Mice were pretreated with control (P4), P5 and P6 peptides (8 μg/g body weight) one day before cancer cells injection. MDA-MB-231/vector and MDA-MB-231/VEGF-C expressing cells stably expressing luciferase were i.v. injected into SCID mice by tail vein injection, and peptides were administered twice weekly for 8 weeks. Lung colonizations were bioluminescently imaged after 8 weeks (**A**) with the mean signal for each group (n=8) indicated (**B**). (**C**) Schematic representation of the activation of VEGFR-3-targeting peptides on cancer progression. Two candidate peptides, P5 and P6 VEGFR-3-targeting peptides inhibit the activation of VEGFR-3 though blocking interaction between VEGFR-3 and VEGF-C, and subsequently abolish VEGFR-3 phosphorylation and further reduce metastasis, drug resistance and TICs formation.

To confirm our *in vitro* findings, we stably transfected a VEGF-C-expressing vector (MDA-MB-231/VEGF-C) or a control vector (MDA-MB-231/vector) into MDA-MB-231 cells for use in experimental metastasis assays. After injection of the indicated cancer cells into mouse tail veins, the control (P4), P5 and P6 peptides were administered twice weekly for 8 weeks, and lung metastatic nodules were then detected with a bioluminescent imaging system. Our data demonstrated that VEGF-C-induced lung metastasis was significantly inhibited by the P5 and P6 peptides in an animal model (Figure 5).

## DICUSSION

Previous experimental and clinical studies have demonstrated that VEGFR-3 plays critical roles in lymphangiogenesis and in the metastatic spread of tumors [12, 27]. VEGFR-3 is activated by its ligand VEGF-C, which results in the phosphorylation of tyrosine residues. Such phosphorylation events are known to regulate receptor kinase activity and signal transduction in cancer cells [14, 15, 28]. VEGFR-3 is therefore one of the most attractive targets for anticancer therapeutics that are designed to restrict cancer progression. In this study, we identified two novel small peptides that exhibit anti-tumor effects, including the suppression of migration, invasion, drug resistance, and TIC formation (Figure 5C). These peptides may be potential candidate for controlling tumor metastasis and chemotherapeutic resistance.

For several decades, the surface receptors of cancer cells have been successfully used as molecular targets for cancer therapy and diagnosis because of the overexpression of these receptors in tumors relative to normal tissues. Considering its key roles in tumor metastasis and lymphangiogenesis, the targeting of VEGFR-3 will be therapeutically significant for human tumors. The activation of the VEGF-C/VEGFR-3 axis has been found to promote the resistance of leukemic cells to chemotherapeutic drugs such as cisplatin, paclitaxel, doxorubicin, and etoposide [10]. In this study, we not only identified a protective effect of VRGFR-3 in solid tumor cells in response to drug treatment but also confirmed the therapeutic effects of VRGFR-3-targeting peptides in the sensitization of cancer cells treated with Taxol and cisplatin. In addition, the VEGFR-3/VEGF-C axis has been shown to maintain the features of TICs that facilitate cancer cells to become more resistant to drugs and have stronger metastatic abilities [24, 29]. We found that the VEGFR-3-targeting peptides decreased the number of VEGF-C/VEGFR-3-induced TIC formation in lung and breast cancer cells. These data suggest that VEGFR-3-targeting peptides have multiple anticancer functions. Thus, the continued studies of VEGFR-3-targeting agents will be essential for developing new therapies that limit the spread of cancer.

Peptides are considered suitable for cancer treatment because of their low molecular weight and easy synthesis. Therapeutic peptides require high specificity, bioavailability, and stability. However, the major limitations of peptides that are used in the human body include fast proteolytic cleavage, a short half-life, and low bioavailability. Significant advances have been achieved through the modification of synthesis strategies, which include PEGylation, lipidization, and multimerization. These modifications decrease renal clearance, enhance water solubility, and improve delivery efficiency. These modifications therefore greatly enhance the stability, half-life, and bioavailability of the peptides, which make them promising solutions for accelerating the translation of peptides from biologically active agents to successful clinical drugs. Recent studies of therapeutic peptides make the peptide-based cancer therapy an attractive approach because without problems with affinity, specificity and safety, peptides may be superior to small molecules and antibodies [30]. Thus, the targeting of surface receptors that are overexpressed in tumor cells by peptides will be one of the most promising approaches for cancer therapy in the future. Currently, more than 70 therapeutic peptides are on the market, and hundreds of peptides are in clinical and preclinical trials. Many pharmaceutical companies are working on the development of bioactive peptides. With advances in modifications, peptides can target cancer in a more ‘natural’ way that is comparable with the endogenous system because it requires high selectivity and potency combined with low toxicity of the peptides themselves and their cleavage products. Accordingly, peptide drugs are truly innovative in pharmaceutical research for many applications. Here we identified novel peptides with significant effects on blocking VEGFR-3, which is important and valuable for further investigations of novel therapeutic strategies in cancer patients.

## MATERIAL and METHODS

### Antibodies and reagents

Anti-human VEGFR-3 antibody and goat anti-mouse rabbit IgG were from Millipore Corp. (Milford, MA). Anti-phospho-VEGFR-3 (Tyr-1063/1068) antibody was from Cell Applications (San Diego, CA). Anti-VEGF-C antibody was from Cell Signaling Technology (Danvers, MA). Anti–α-tubulin antibody, cisplatin and Taxol were from Sigma (St. Louis, MO). Recombinant human VEGF-C (Cys156Ser), bFGF and EGF protein were purchased from R&D Systems (Minneapolis, MN). Anti–phospho–src antibody and Anti–src antibody were from GeneTex (Hsinchu City, Taiwan, R.O.C). Biotin-labeled goat anti–rabbit IgG, tetramethyl benzidine (TMB) and streptavidin-HRP were from Kirkegaard & Perry Laboratories (Gaithersburg, MD). Soluble VEGFR-3 was from Angio-Proteomie (Boston, MA).

### Cell culture

Lung adenocarcinoma cell lines (CL1-5) were established at the National Health Research Institutes laboratory [31]. Lung cancer cell lines (H322 and H460) and breast cancer cell lines (BT474) were obtained from ATCC and grown in RPMI-1640 medium supplemented with 10% FBS. The lung cancer cell line (A549), breast cancer cell lines (MDA-MB-231, MCF-7, HS578T) and the human embryonic kidney 293T cell line were obtained from ATCC and cultured in DMEM/F12 supplemented with 10% FBS. The breast cancer cell lines HBL100 and MDA-MB-361 were obtained from ATCC and grown in DMEM medium that was supplemented with 10% FBS. Human embryonic kidney 293T/VEGFR-3 cells, MDA-MB-231/vector and MDA-MB-231/VEGF-C cells were cultured in DMEM/F12 containing 10% FBS and blasticidin (2 μg/ml). Normal human lymphatic endothelial cells (No. C-12218) were purchased from PromoCell (Heidelberg, Germany) and cultured in endothelial cell growth medium MV2.

### Screening a 7-mer phage display library with soluble VEGFR-3

The procedure for screening the phage display library was modified according to the manufacturer (New England Biolabs, Ipswich, MA). Culture dishes were coated with human soluble VEGFR-3 (sVEGFR-3). sVEGFR-3 was added at 100 ng/mL and incubated at 4°C for 24 hours before blocking with phosphate buffer containing 1% bovine serum albumin (BSA) for 1 hour at 37°C. The phage library containing 2 × 10^9^ clones was sequentially added to non-bait-coated dishes for preabsorption. In each case, the library was shaken gently at room temperature for 1 hour. Finally, the preabsorbed library was applied to sVEGFR-3–coated dishes for further screening. After thorough washing, plate-bound phage clones were eluted with elution buffer [0.22 mol/L glycine-HCl (pH 2.2)] and immediately neutralized. Four rounds of selection were done, after which individual plaques were picked at random and subjected to analysis by phage ELISA and DNA sequencing following amplification in *E. coli* ER2537.

### Competition assays of synthesized peptides to rhVEGF-C

Soluble VEGFR-3 (sVEGFR-3) (250 ng/mL) (Angio-Proteomie, Boston, MA) was immobilized on 96-well plates and blocked with phosphate buffer containing 1% BSA for 1 hour at room temperature. In competition experiments, peptides were incubated with the VEGFR-3-coated plates for 6 hours at room temperature. After incubation, human recombinant VEGF-C protein (rhVEGF-C, 300 ng/mL, 100 μL) was added directly to the wells without removal of the peptides and then incubated at room temperature for 6 hours. The plates were thoroughly washed with 0.1% BSA/PBS buffer (pH 8.5), and anti–VEGF-C Ab was added at 4°C for 24 hours. Biotin-conjugated goat anti-rabbit IgG antibody was added for 1 hour. HRP-conjugated streptavidin was then added for 30 minutes. The free streptavidin conjugate was washed away and freshly prepared substrate solution (TMB) was then added to each well. The reaction was allowed to stand for 15 minutes, and the color development was stopped by the addition of H_3_PO_4_ (1.0 mol/L). The absorbance at 450 nm was read with a reference wavelength of 650 nm (A450/650).

### VEGFR-3-Kinase Assay

Kinase assays were performed as previously described [32]. Briefly, human soluble protein kinase VEGFR-3 (sVEGFR-3) that was expressed in insect cells was used for the kinase assays. The candidate peptides were preincubated with 0.1 μg/ml of enzyme in modified HEPES buffer pH 7.4 for 15 minutes at 37°C. The reaction was initiated by addition of 0.2 mg/ml poly(Glu:Tyr), 10 μM ATP and 0.25 μCi [γ^32^P]ATP for another 30 minute incubation period and terminated by further addition of 3% H_3_PO_4_. Aliquots were removed and counted to determine the amount of [^32^P] Poly(Glu:Tyr) that formed. Compounds were screened at 10 μM. All peptides were analyzed by Eurofins Panlabs Taiwan Ltd.

### Western blot analysis

Cells were incubated in serum-free DMEM/F12 for 24 hours before treatment with or without rhVEGF-C (100 ng/mL). Cells were lysed in RIPA buffer [Tris-HCl (50 mM; pH 7.5), NaCl (120 mM), NP-40 (0.5%), Na_3_VO_4_ (200 mM), EDTA (100 mM), sodium deoxycholate (0.5%), SDS (1%)] for 10 minutes on ice. An equal quantity of protein from the cell lysates was resuspended in gel sample buffer, resolved by 10% SDS-PAGE, and transferred to polyvinylidene difluoride membrane membranes (Millipore Corp., Milford, MA). After blocking, membranes were incubated with specific primary antibodies and corresponding secondary antibodies. Immunoreaction signals were visualized by an enhanced chemiluminescence detection system (PerkinElmer Health Sciences, Waltham MA).

### Kinase receptor activation enzyme-linked immunosorbent assay (KIRA-ELISA)

H928 lung cancer cells (2×10^5^) were cultured in 24-well plates and serum-starved for 24 hours. Cells were pretreated with candidate peptide for 30 minutes and then treated with rhVEGF-C for 15 minutes. Cells were solubilized in RIPA lysis buffer for 60 minutes. Another 85 μl of cell lysate was transferred to an ELISA well that was coated with VEGFR-3 and incubated for 2 hours. After the plates were washed, biotinylated-4G10 antibody (500 ng/ml, 100 μl) was added and incubated for 2 hours. After the plates were washed, HRP-conjugated streptavidin was then added for 30 minutes. The free avidin conjugate was washed away and freshly prepared substrate solution (TMB) was then added to each well. The reactions were allowed to proceed for 10 minutes, and the color development was stopped by the addition of H_3_PO_4_ (1.0 mol/L). The absorbance at 450 nm was read with a reference wavelength of 650 nm (A450/650).

### Cell migration/invasion assay

Boyden chamber cell migration assays were performed using uncoated transwells and matrigel-coated transwells with membranes (8 μm pore-size) (Becton Dickinson, Mountain View, CA). The chambers were inserted into 24-well culture plates that contained DMEM/F12 with 10% fetal bovine serum. The cells were pretreated with peptide (100 μM) in serum-free DMEM/F12 medium and were loaded into the transwells. After 24 hours, non-migrated cells were removed with a cotton swab and the cells were fixed with methanol for 15 min and stained with crystal violet. The migrated cells were quantified by blind counting of the migrated cells on the lower surface of the membrane from at least 10 fields per chamber using a 20X objective. All migration and invasion assays were normalized to the proliferation rate.

### Cell death by DNA flow cytometric assay

Briefly, cells were treated with 20 nM Taxol or 10μM cisplatin and incubated for 48 hours. Samples were prepared and analyzed as previously described [33].

### Cell viability evaluation by trypan blue dye exclusion assay

Briefly, cells were harvested using trypsin and stained with 0.4% trypan blue dye (Sigma-Aldrich). Trypan blue-positive and -negative cells were counted using a hemacytometer (Hausser Scientific, Horsham, PA) under a phase-contrast microscope (Fisher Scientific, Pittsburgh, PA). The results of each assay were expressed in terms of the percentage of dead cells relative to the total number of cells. Individual experiments were performed in triplicate.

### Cell viability by MTT assay

A549 lung cancer cells (3×10^3^) were cultured in 96-well plates and serum-starved for 24 hours. Cells were treated with the candidate peptides (200 μM) for 4 hours and were treated with rhVEGF-C (100 ng/mL) for 6 hours in serum-free medium. After 48 hours, MTT (5 mg/ml) was added for 3 hours and the precipitates were dissolved in DMSO. The absorbance at 570 nm was read with a reference wavelength of 630 nm (A570/630).

### Sphere-forming assay

A549 and MCF-7 cells were serum-starved for 24 hours. The cells were pretreated with candidate peptides (200 μM) in the presence or absence of rhVEGF-C for 24 hours. The cells were trypsinized and mechanically dissociated to generate single cells that were then seeded at a density of 1×10^3^ cells/ml in serum-free DMEM/F12 medium (Invitrogen, Carlsbad, CA) that was freshly supplemented with epidermal growth factor (20 ng/ml), basic fibroblast growth factor (20 ng/ml) (R&D Systems, Minneapolis, MN), 1X B27 supplement, 1X N2 supplement (Invitrogen, Carlsbad, CA) in the presence or absence of rhVEGF-C (200 ng/mL) and candidate peptides (200 μM) in non-adherent plates for 10 days. Quantification of sphere formation was determined by measuring the percentage of 5000 spheres.

### Isolation of RNA and reverse transcription

Total RNA was isolated after A549 spheres had grown for 10 days by using Trizol (Invitrogen, Carlsbad, CA), and cDNA were synthesized with Oligo dT (Thermo Scientific, Brookfield, WI). The specific primers for *SOX2* were 5'-TACAACATGATGGAGACG-3' and 5'-GCGTGTACTTATCCTTCTTC-3'. The reaction was cycled at 95°C for 40 seconds, then 56°C for 40 seconds, and then 72°C for 30 seconds for 30 cycles followed by a final extension of 72°C for 5 minutes.

### Animal Protocols

All animal work was performed in accordance with protocols that were approved by the Institutional Animal Care and Use Committee of the National Health Research Institutes. All of the animal experiments were performed on 6- to 8-week-old CB-17 severe combined immunodeficient (SCID) female mice (supplied by LASCO, Taiwan) with eight animals per group. The control (P4), P5 and P6 peptides were pretreated with 8 μg/g body weight dissolved in 50 μl PBS and i.v. injected into mice one day before cancer cell injection. For experimental metastasis assays, 2.5 × 10^5^ viable cells were resuspended in 0.1 mL of PBS and introduced into the circulation via tail vein injection. For the treatment, peptides were administered (8 μg/g body weight) twice weekly for 8 weeks. The luciferase-based, non-invasive bioluminescent imaging and analysis were performed with the Xenogen IVIS-200 system (Xenogen, Alameda, CA, USA).

## SUPPLEMENTARY FIGURES AND METHODS


