# Supplementary material for: Novel peptides suppress VEGFR-3 activity and antagonize VEGFR-3-mediated oncogenic effects
Source: Oncotarget. 2014 May 24;5(11):3823–35. doi: 10.18632/oncotarget.1709 (PMC4116523; doi:10.18632/oncotarget.1709)
Supplement: Supplementary file 1 [file oncotarget-05-3823-s001.pdf]

SUPPLEMENTARY FIGURES AND METHODS

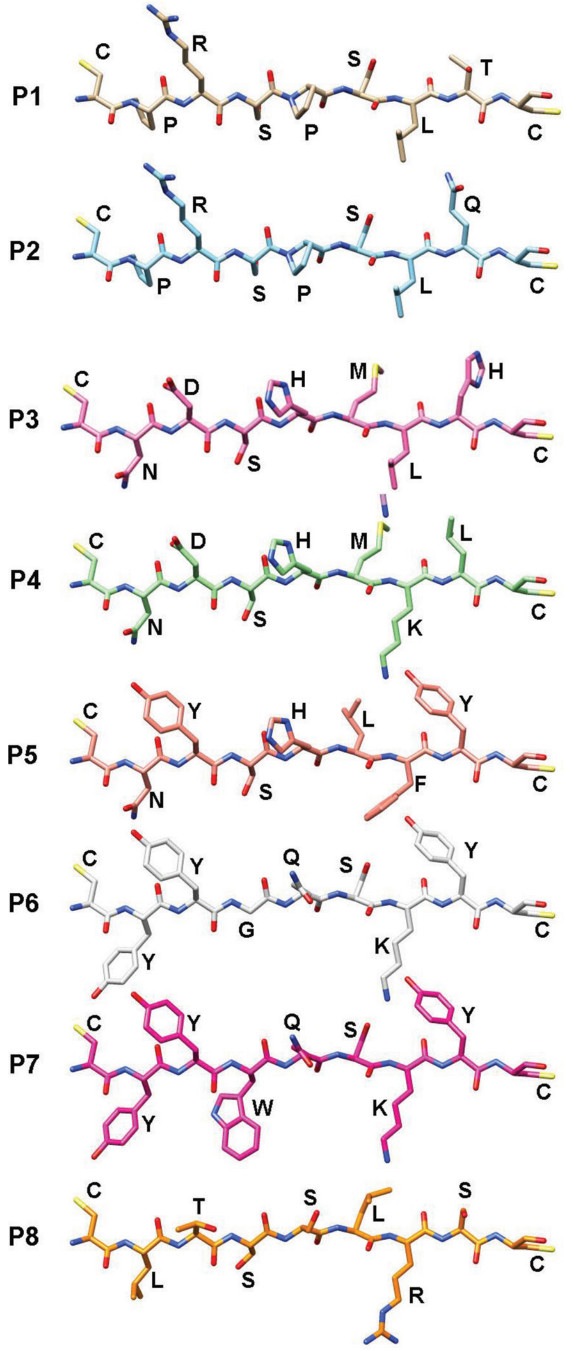

Figure S1: The peptide residues were shown as stick models with carbon atoms in different colors. From upper to lower, we presented structures of P1 to P8 peptides.

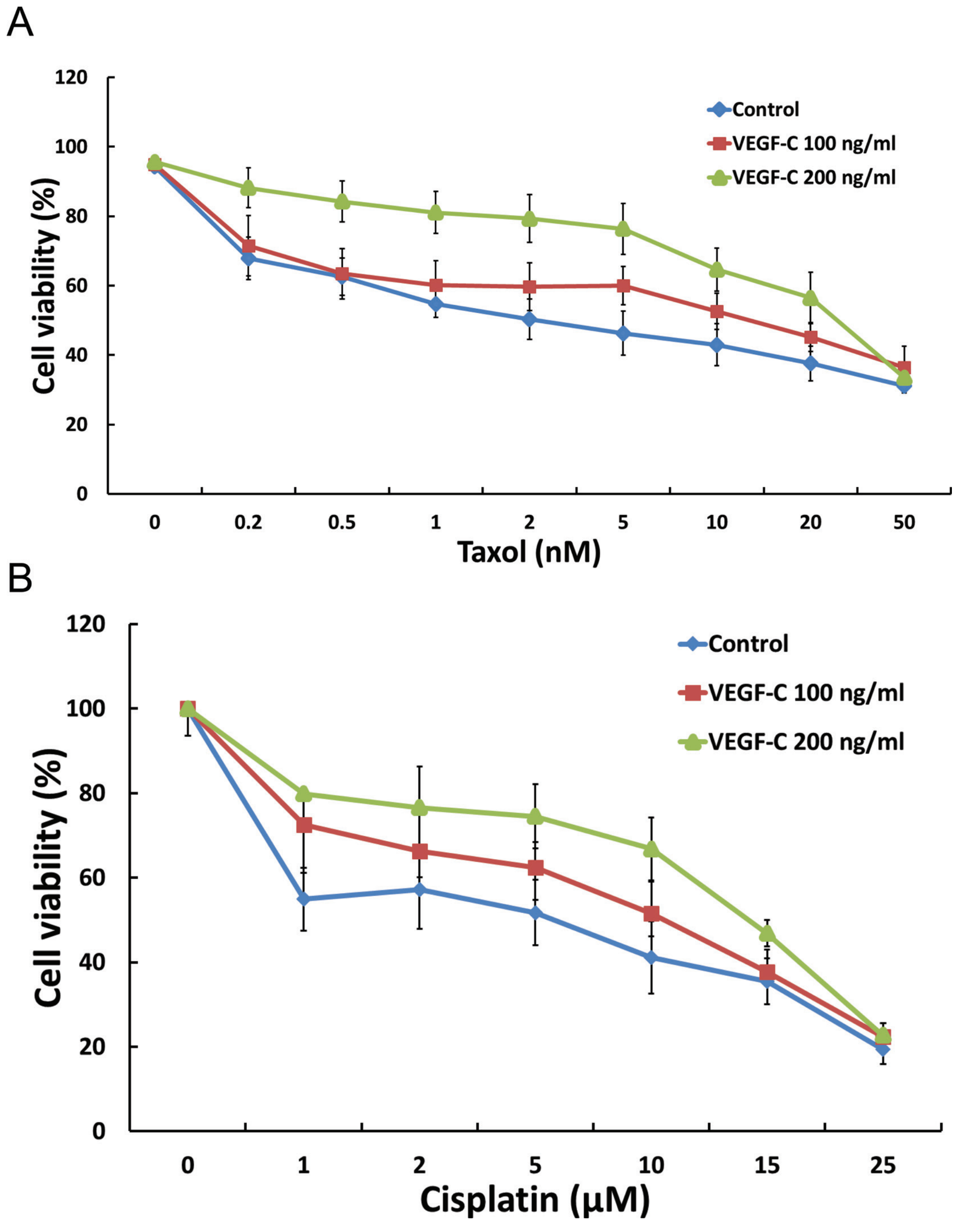

Figure S2: VEGF-C protected lung cancer cell from anti-cancer drug-induced cell death. MTT assay was performed to analysis the cell viability of A549 cells by co-treating with Taxol **(A)** or cisplatin **(B)** in the presence or absence of rhVEGF-C. **(A)** A549 cells were starved for 24 hours in 96 wells before rhVEGF-C treatment (100 ng/ml and 200 ng/ml). After rhVEGF-C treatment, cells were treated with indicated concentration of Taxol. Survived cells were determined by MTT assay. **(B)** A549 cells were serum-starved for 24 hours in 96 wells before rhVEGF-C treatment (100 ng/ml and 200 ng/ml). After rhVEGF-C treatment, cells were treated with indicated concentrations of cisplatin. Survived cells were determined by MTT assay. Each assay was done in three separate experiments; bars, SD.

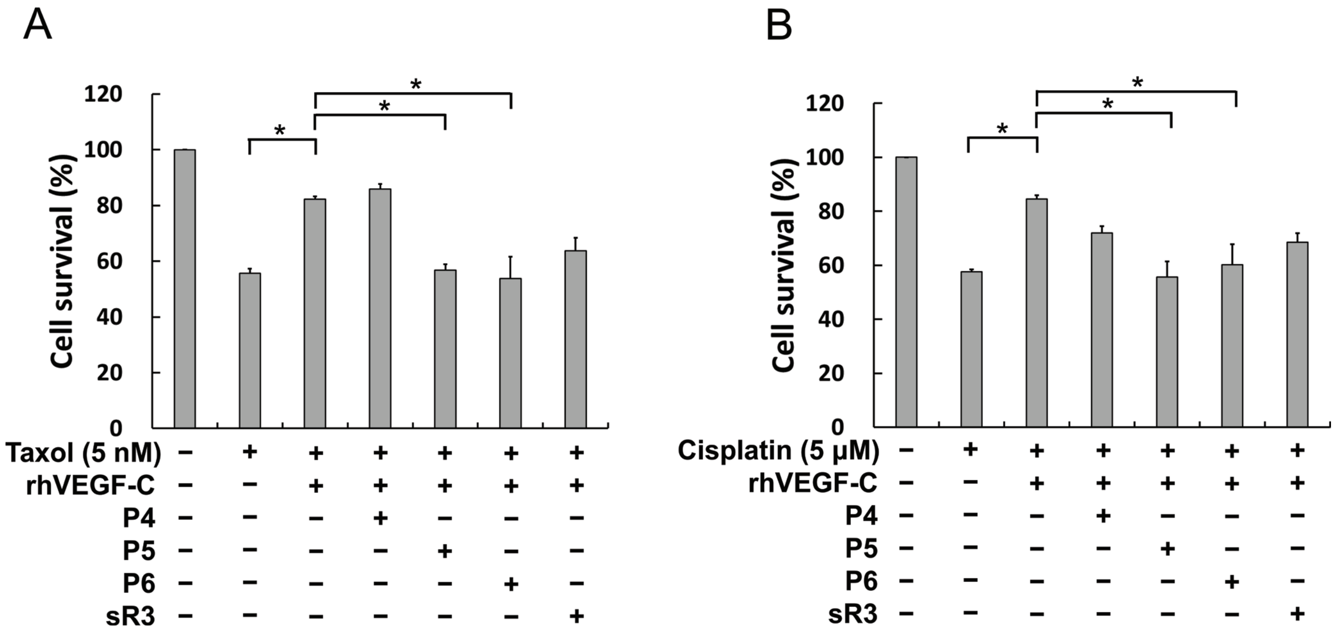

Figure S3: P5 and P6 peptides inhibit VEGF-C-induced drug resistance by MTT assay. Taxol **(A)** and cisplatin **(B)** were treated with the indicated peptides and rhVEGF-C in A549 cells. The cell survival rates were measured by MTT assay. Columns, mean of three independent experiments; bars, SD. *, $p < 0.05$ statistically significant decrease compared with the corresponding control value.

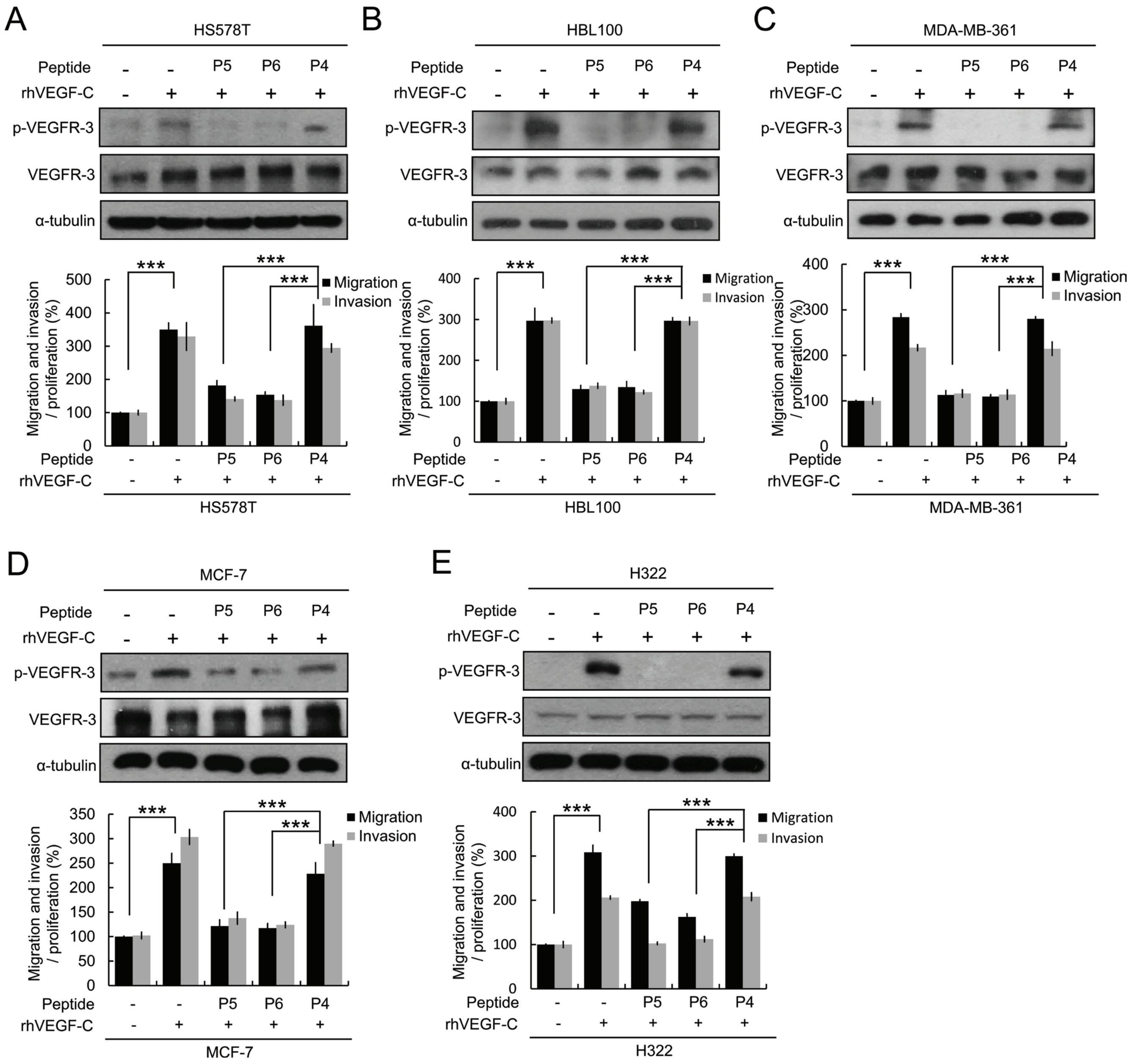

Figure S4: P5 and P6 peptides inhibit VEGF-C/VEGFR-3 signaling and decrease cancer cells migration and invasion. Effects of control (P4), P5 and P6 peptides on VEGF-C-induced VEGFR-3 activation was analyze by western blot analysis (upper) and the ability of migration and invasion (lower) in HS578T **(A)**, HBL100 **(B)**, MDA-MB-361 **(C)**, MCF-7 **(D)** and H322 **(E)** cells. Columns, mean of three independent experiments; bars, SD. ***, $p < 0.001$ statistically significant decrease compared with the corresponding control value.

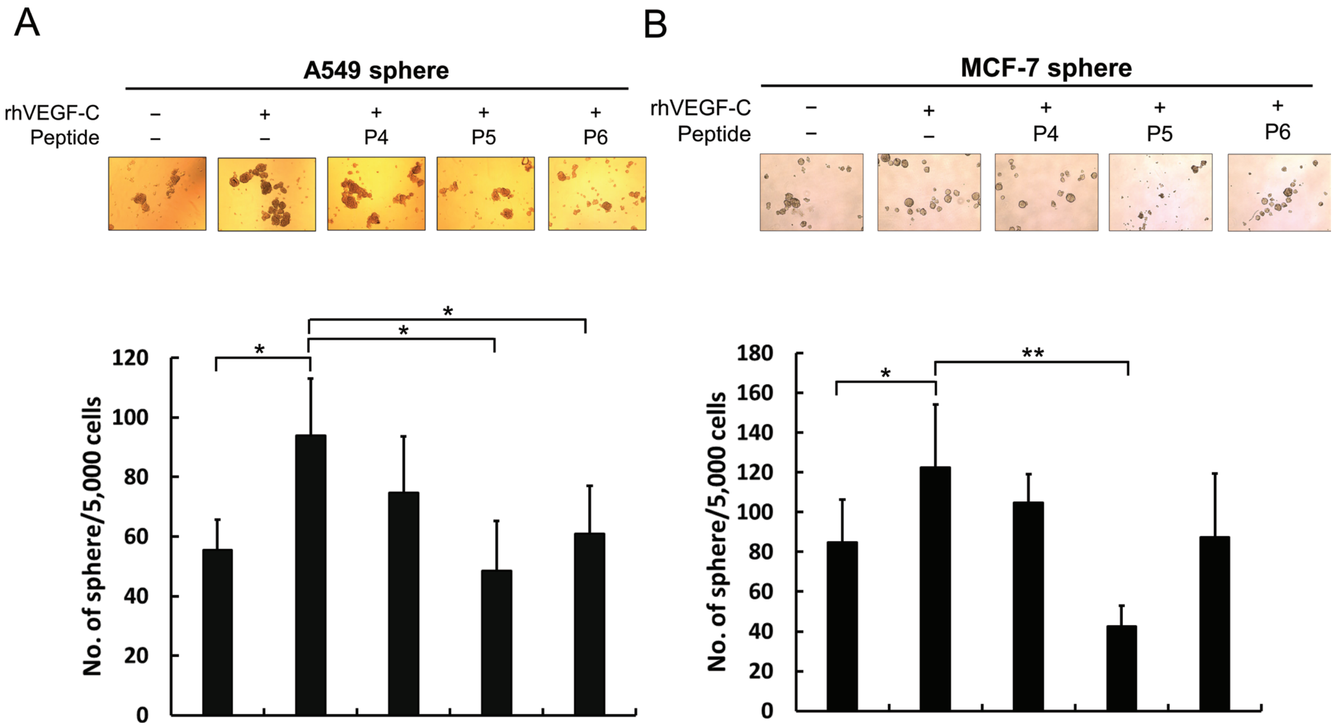

Figure S5: P5 and P6 peptides decrease sphere formation of cancer cells. Effects of control (P4), P5 and P6 peptides on VEGF-C-induced tumor initiating cells (TICs) formation. Sphere-forming assay was perform in A549 **(A)** and MCF-7 **(B)** cells treated with indicated peptides and rhVEGF-C. Columns, mean of three independent experiments; bars, SD. *, $p < 0.05$; **, $p < 0.01$ statistically significant decrease compared with the corresponding control value.

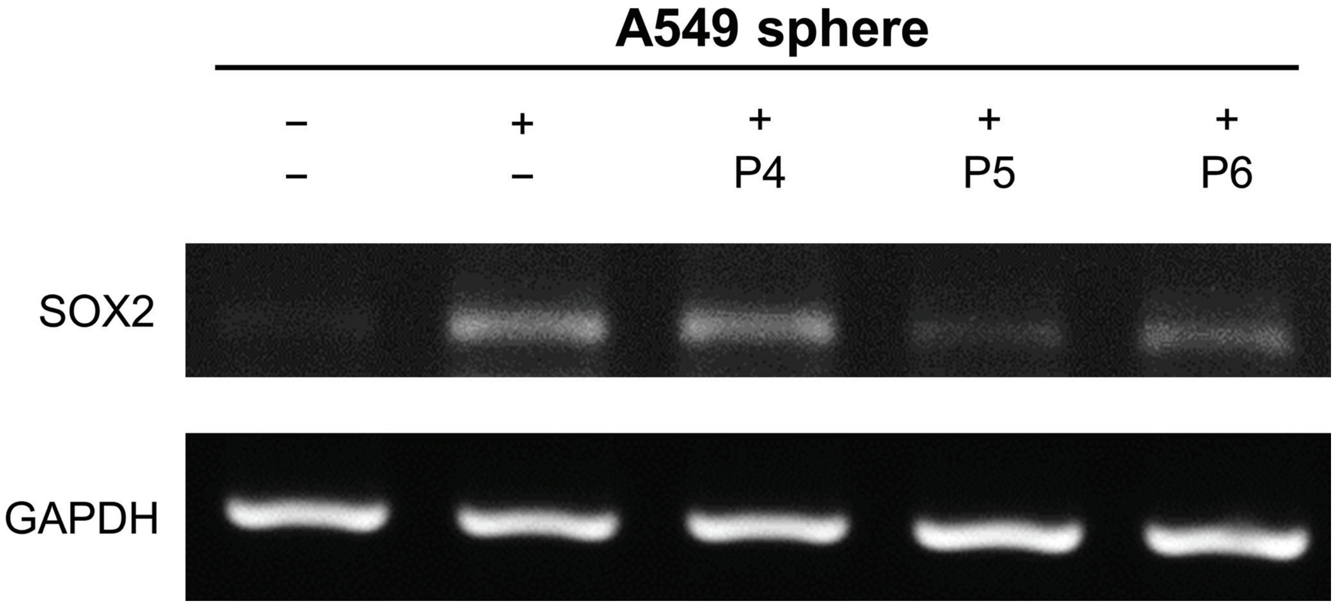

Figure S6: P5 and P6 peptides significantly decrease VEGF-C-induced cancer stem cell marker. Effects of P5 and P6 peptides on mRNA expression of SOX2. Total RNA were harvested for analysis of SOX2 mRNA by RT-PCR.
